# Higher Muscle Mass Implies Increased Free-Thyroxine to Free-Triiodothyronine Ratio in Subjects With Overweight and Obesity

**DOI:** 10.3389/fendo.2020.565065

**Published:** 2020-09-29

**Authors:** Roberta Zupo, Fabio Castellana, Rodolfo Sardone, Luisa Lampignano, Silvia Paradiso, Vito Angelo Giagulli, Vincenzo Triggiani, Luigi Di Lorenzo, Gianluigi Giannelli, Giovanni De Pergola

**Affiliations:** ^1^ Population Health Unit – “Salus in Apulia Study”, National Institute of Gastroenterology “Saverio de Bellis”, Research Hospital, Bari, Italy; ^2^ Clinical Nutrition Unit, Department of Biomedical Science and Human Oncology, University of Bari, School of Medicine, Policlinico, Bari, Italy; ^3^ Section of Internal Medicine, Geriatrics, Endocrinology and Rare Disease, Interdisciplinary Department of Medicine, School of Medicine, University of Bari, Bari, Italy; ^4^ Dipartimento di Medicina Interna e Medicina Pubblica, Sezione di Medicina del Lavoro “E.C. Vigliani”, University of Bari, Policlinico, Bari, Italy; ^5^ Scientific Direction, National Institute of Gastroenterology “Saverio de Bellis”, Research Hospital, Bari, Italy

**Keywords:** obesity, body composition, skeletal muscle mass, thyroid hormone, Italy

## Abstract

Thyroid hormones control both metabolic pathways and body composition, whereas little knowledge is available about the possible influence of skeletal muscle mass (MM) on thyroid hormone metabolism and circulating levels. This was a cross-sectional study conducted at the Population Health Unit of the National Institute of Gastroenterology IRCCS “S. de Bellis” (Italy) and investigating the extent to which skeletal MM affects thyroid function in obesity. Two hundred twenty-seven consecutive healthy volunteers (155 women and 72 men) with overweight and obesity (BMI ≥ 25 kg/m^2^) and taking no medication or supplement were assessed for hormone, metabolic and routine laboratory parameters. Body composition parameters were collected by using bioelectrical impedance analysis (BIA). MM was directly related to the body mass index (BMI), waist circumference (WC), insulin, triglycerides, uric acid and free-triiodothyronine (FT3) serum levels, FT3 to the free-thyroxine (FT4) ratio, and insulin-resistance (HOMA-IR), and inversely related to age, total, and HDL-cholesterol serum levels. Multiple regression models confirmed the relationship between MM and the FT3 to FT4 ratio, independently of age, BMI, TSH, triglycerides, and insulin serum levels. The same analyses run by gender showed that this relationship maintained significance only in men. Increased skeletal MM in obesity results in improved thyroid activity mediated by increased T4 conversion to T3, and higher FT3 circulating levels, particularly in men. In conclusion, preserving a greater skeletal MM in obesity helps to enhance thyroid activity.

**Clinical Trial Registration:**

ClinicalTrials.gov, identifier NCT04327375.

## Introduction

In recent decades, obesity rates have more than doubled, to the extent that nearly one third of the world population is now classified as overweight or obese (1). Despite its debatable sensitivity, the body mass index (BMI) is still the parameter most commonly used to define obesity in clinical practice. According to the latest data from the Global Burden of Disease Study (GBD), a high BMI showed the greatest relative increase in exposure since 1990, and is thus actually ranked among the top five risk factors in terms of attributable deaths and disability-adjusted life-years (DALYs) (2). Obesity is a multifactorial condition characterized by an excessive accumulation of body fat resulting from the interaction between environmental and genetic factors (3, 4). Among lifestyle contributory factors, dietary habits play a leading role in weight gain in terms of energy balance. Indeed, a chronic positive energy balance resulting from an excessive dietary energy intake versus energy expenditure leads to a form of metabolic failure deriving from the inability of oxidative processes to cope with the surplus calories intake (5).

Thyroid hormones play an important role in regulating many metabolic pathways, since they are involved in accelerating thermogenesis and oxygen consumption, as well as controlling lipid and glucose metabolism. On this basis, hypothyroidism is usually associated with a decreased thermogenesis and metabolic rate, inducing a modest weight gain, whereas hyperthyroidism leads to weight loss despite an increased appetite and elevated metabolic rate (6).

Obesity is responsible for a moderate increase in serum thyrotropin (TSH) concentrations, due to insulin resistance and elevated leptin levels (7, 8), but also for slightly above-normal free plasma levels of triiodothyronine (T3). This latter is widely acknowledged as a compensatory mechanism reducing further weight gain, since higher thyroid hormone concentrations physiologically increase resting energy expenditure (REE) (9, 10).

To date, the large volume of research investigating body modifications in obesity is consistent with the conviction that gaining weight elicits several adaptations in all body compartments, especially fat storage (11). Besides the increase in fat mass, obesity also induces a simultaneous growth of fat-free and muscle mass (MM) (12). In particular, an increased lower limb skeletal MM has been described as a way to compensate for the increased fat mass, in order to support standing and ambulation (13). Thus, since the skeletal muscle is a major site for thermogenesis, glucose utilization and lipid oxidation, the possibility of a close interplay between thyroid gland function, and MM is quite viable.

Moreover, it should be remembered that human skeletal muscle is the site of type II iodothyronine deiodinase activity, converting T4 to T3 (14, 15). Given that obesity is characterized by an increase of both MM and FT3 levels, it may well be that a higher MM is responsible for higher type II iodothyronine deiodinase, thus increasing free T3 production and circulating hormone levels.

To the best of our knowledge, studies clarifying thyroid performance in response to body MM changes in obesity are lacking. The purpose of this study was to examine the relationship between skeletal MM and thyroid hormone levels among subjects with overweight and obesity, focusing on FT4 conversion to FT3, as well as on changes in FT3 serum levels.

## Material and Methods

### Study Patients

From January 2018 to December 2019, 227 consecutive patients (155 women and 72 men, aged 42.4 ± 13.3 years) were recruited at the Population Health Unit of the National Institute of Gastroenterology “S. de Bellis”, Research Hospital, Castellana Grotte, Italy. In the power analysis, to detect a variance of the FT3/FT4 ratio parameter between the reference population (10) compared to our population used in the general linear model, a sample size at least of 19 was required, with 90% power for a two-sided alpha error probability of 5%.

All data collection was done at the baseline examination. Inclusion criteria were overweight or obesity (BMI ≥ 25 kg/m^2^), and taking no supplements or medication, including oral contraceptives or medicines for osteoporosis. Exclusion criteria were any history of endocrinological diseases (diabetes mellitus, hypo or hyperthyroidism, hypopituitarism, etc.), chronic inflammatory diseases, stable hypertension, angina pectoris, stroke, transient ischemic attack, heart infarction, atrial fibrillation, congenital heart disease, any malignancies, renal and liver failure and inherited thrombocytopenia.

The study protocol (ClinicalTrials.gov Identifier: NCT04327375) met the principles of the Declaration of Helsinki and was approved by the Ethics Committee of the National Institute of Gastroenterology “S. De Bellis” Research Hospital (Castellana Grotte, Italy). All participants gave informed consent prior to enrollment in accordance with the Helsinki Declaration of 1964 and subsequent revisions.

### History, Objective Examination, and Fluid Biomarkers Collection

At baseline, subjects were closely examined for hormone, metabolic and routine biochemistry parameters. A brief interview, including questions on medical history and lifestyle, was conducted by a physician. Extemporaneous outpatients diastolic (DBP) and systolic blood pressure (SBP) was determined in a sitting position after at least a 10-min rest, a minimum of three different times, using an OMRON M6 automatic Blood Pressure monitor. Blood samples were drawn between 08:00 h and 09:00 h after overnight fasting. Fasting blood glucose, insulin, total cholesterol, high- and low-density lipoprotein (HDL, LDL) cholesterol, and triglycerides serum levels were assayed. Serum insulin concentrations were measured by radioimmunoassay (Behring, Scoppito, Italy) and all samples were analyzed in duplicate. Plasma glucose was determined using the glucose oxidase method (Sclavus, Siena, Italy), while the concentrations of plasma lipids (triglycerides, total cholesterol, HDL cholesterol) were quantified by automated colorimetric method (Hitachi; Boehringer Mannheim, Mannheim, Germany). LDL cholesterol was calculated using the Friedewald equation.

TSH, FT3, and FT4 serum concentrations were measured by a competitive luminometric assay based on the SPALT (solid-phase antigen luminescence technique) principle (LIAISON FT3, FT4, TSH, DiaSorin, Saluggia, Italy). Serum uric acid was measured by the URICASE/POD method implemented in an autoanalyzer (Boehringer Mannheim, Mannheim, Germany). Insulin resistance was assessed using the Homeostasis Model Assessment for Insulin Resistance (HOMA-IR) (16).

The metabolic syndrome was assessed according to IDF criteria (17), consisting of abdominal obesity (WC ≥ 94 cm for men and ≥ 80 cm for women) together with at least 2 of the following parameters: hypertension (SBP ≥ 130 mm Hg and/or DBP ≥ 85 mm Hg) or a history of antihypertensive drugs, hypertriglyceridemia (≥ 150 mg/dl) or treatment for this disorder, low HDL-C (< 40 mg/dl in men and < 50 mg/dl in women) or treatment for this disorder, and high fasting plasma glucose ≥ 100 mg/dl) or a diagnosis of T2DM.

### Anthropometric and Bioelectrical Impedance Analysis

Clinical procedures were carried out by two certified nutritionists (R.Z. and F.C.) both previously trained to equivalent measuring performances. All anthropometric measurements were taken with participants wearing lightweight clothing and no shoes. Variables were all collected at the same time between 7:00 and 10:00 a.m., following an overnight fast.

Height was measured to the nearest 0.5 cm using a wall-mounted stadiometer (Seca 711; Seca, Hamburg, Germany). Body weight was determined to the nearest 0.1 kg using a calibrated balance beam scale (Seca 711; Seca, Hamburg, Germany). BMI was calculated by dividing body weight (kg) by the square of height (m^2^) and classified according to World Health Organization criteria for normal weight (18.5–24.9 kg/m^2^), overweight (25.0–29.9 kg/m^2^), grade I obesity (30.0–34.9 kg/m^2^), grade II obesity (35.0–39.9 kg/m^2^), and grade III obesity (≥40.0 kg/m^2^) (18). Waist circumference (WC) was measured at the narrowest part of the abdomen, or in the area between the tenth rib and the iliac crest (minimum circumference).

Bioelectrical Impedance Analysis (BIA) was performed using a single-frequency bioimpedance analyzer (BIA‐101 analyzer, 50‐kHz frequency; Akern Bioresearch, Florence, Italy). The instrument is routinely checked with resistors and capacitors of known values. In accordance with the European Society of Parenteral and Enteral Nutrition (ESPEN) guidelines (19), all participants were examined supine with their legs spread slightly apart, and had refrained from eating, drinking, and exercising for six hours and drunk no alcohol within 24 h before examination. Shoes and socks were removed and contact areas scrubbed with alcohol prior to electrode placement. Electrodes (BIATRODES Akern, Florence, Italy) were placed proximal to the phalangeal-metacarpal joint on the dorsal surface of the right hand and distal to the transverse arch on the superior surface of the right foot. Sensor electrodes were placed at the midpoint between the distal prominence of the radius and ulna of the right wrist, and between the medial and lateral malleoli of the right ankle (20). All measurements were performed by a senior nutritionist (SP) under strictly standardized conditions. Whole‐body impedance vector components, resistance (R, Ω) and reactance (Xc, Ω), were derived and recorded when stable.

Then, according to age, gender, weight and height of each subject, body composition parameters were obtained using the software provided by the manufacturer, which included validated (19) predictive equations for total body water (TBW, L), extracellular water (ECW, L), intracellular water (ICW, L), fat-free mass (FFM, kg), MM (kg), Appendicular skeletal muscle mass (ASMM, kg), and body cell mass (BCM, kg). Phase angle (PhA, °) values, derived from the ratio of reactance versus electric resistance, were collected as well. A more detailed description of all principles applied to derive bioimpedance measurements has been previously made elsewhere (21, 22).

### Statistics

We performed statistical analysis of baseline variables as the mean ± standard deviation (SD) for continuous variables and proportion (%) for the frequency of categorical variables. The normality of distribution was assessed for each variable using Shapiro’s test. Wilcoxon sum rank test and independent samples t-test were performed on the basis of methodological suitability, to assess differences of collected variables between groups. A Spearman’s correlation matrix was applied to all continuous biochemical and anthropometrics variables in order to assess the presence of significant correlations. P-values less than or equal to 0.05 were considered statistically significant, with 95% confidence intervals. Three different linear regression models were performed on the FT3/FT4 ratio as dependent variable and MM as regressor, as follows: 1) raw model; 2) partially adjusted model including age, BMI, TSH, insulin, and triglycerides levels as confounders; and 3) fully adjusted model adding gender to the previous parameters. Fully adjusted model performed by applying the previous parameters separately in women and men. The selection of confounders was made considering both the univariate significance with the FT3/FT4 ratio and major covariates such as age, BMI, and gender. The principle for structuring each model was derived from the assumption of a key role of gender in affecting body composition, as well as of an unbalanced distribution of the sample. Statistical analyses were performed using RStudio software, Version 1.2.5001 (RStudio, Inc., Boston, MA, USA).

## Results

There was a greater proportion of women in the whole sample (68.3%). Median age for both genders was around 42 years (range 18–72 years). The mean BMI was 33 ± 5.38 kg/m^2^. Metabolic syndrome showed a higher prevalence in men (40.3%), although a minority of the whole sample was affected (N = 60 of 227 subjects). **Table 1** summarizes the general, anthropometric, hormone, metabolic, and routine biochemistry parameters of the enrolled subjects expressed as mean ± SD and subdivided by gender. Men showed significantly higher cell and lean mass parameters (PhA, BCM, FFM, MM, ASMM), body fluids (ECW, ICW, TBW), WC, systolic and diastolic BP, biomarkers of glucose metabolism (FBG, insulin, HOMA-IR), triglycerides, uric acid, FT3 serum levels and FT3 to FT4 ratio (all showing a P value less than 0.02). TSH and HDL-cholesterol serum levels were significantly higher in women (P = 0.043 and P < 0.01, respectively).

**Table 1:** Characteristics of the whole sample (N = 227), subdivided by gender.

	Women	Men	*P* Value*
Age (years)	42.155 ± 13.054	43.097 ± 14.139	0.788
PhA (°)	6.069 ± 0.706	6.686 ± 0.629	**<0.01**
BCM (Kg)	27.155 ± 3.7	39.844 ± 5.888	**<0.01**
FM (Kg)	34.995 ± 10.848	32.719 ± 13.415	0.072
FFM (Kg)	49.806 ± 7.107	70.31 ± 9.877	**<0.01**
TBW (L)	36.967 ± 4.023	51.889 ± 7.29	**<0.01**
ECW (L)	16.917 ± 2.026	22.096 ± 2.993	**<0.01**
ICW (L)	19.942 ± 2.867	29.446 ± 4.298	**<0.01**
MM (Kg)	23.577 ± 3.427	32.392 ± 5.071	**<0.01**
ASMM (Kg)	20.559 ± 2.906	27.408 ± 4.36	**<0.01**
BMI (Kg/m^2^)	32.798 ± 5.145	33.523 ± 5.877	0.52
WC (cm)	104.548 ± 10.855	115.153 ± 13.471	**<0.01**
SBP (mmHg)	126.568 ± 16.03	135.153 ± 16.187	**<0.01**
DBP (mmHg)	80.948 ± 10.889	87.528 ± 12.369	**<0.01**
Triglycerides (mg/dl)	92.523 ± 44.279	138.236 ± 84.746	**<0.01**
HDL Cholesterol (mg/dl)	57.439 ± 14.887	44.958 ± 10.507	**<0.01**
LDL Cholesterol (mg/dl)	123.806 ± 35.638	123.778 ± 31.629	0.688
Total Cholesterol (mg/dl)	199.2 ± 40.178	193.681 ± 35.673	0.597
FBG (mg/dl)	87.723 ± 10.898	92.681 ± 13.994	**0.017**
Insulin (UI/ml)	11.628 ± 7.327	16.406 ± 15.067	**0.004**
HOMA-IR	2.524 ± 1.673	3.852 ± 3.939	**0.001**
Metabolic Syndrome	31 (20.0)	29 (40.3)	**0.001^χ2^**
Uric Acid (mg/dl)	4.053 ± 1.117	5.662 ± 1.393	**<0.01**
TSH (mU/L)	2.023 ± 1.097	1.733 ± 0.962	**0.043**
FT3 (pg/ml)	2.953 ± 0.415	3.113 ± 0.328	**0.002**
FT4 (pg/ml)	10.327 ± 1.395	10.271 ± 1.327	0.812
FT3 to FT4 ratio	0.29 ± 0.049	0.308 ± 0.049	**0.01**

Wilcoxon sum rank test*, Chi-squared ^χ2^.

Statistical significance shown in bold.

*Numeric variables are shown as mean ± SD and categorical variables as proportion (%).

PhA, Phase Angle; BCM, Body Cell Mass; FM, Fat Mass; FFM, Free-Fat Mass; TBW, Total Body Water; ECW, Extracellular Water; ICW, Intracellular Water; MM, Skeletal Muscle Mass; ASMM, Appendicular Skeletal Muscle Mass; BMI, Body Mass Index; WC, Waist Circumference; SBP, Systolic Blood Pressure; DBP, Diastolic Blood Pressure; FBG, Fasting Blood Glucose; HOMA-IR, Homeostasis Model Assessment for Insulin Resistance); TSH, Thyroid-Stimulating Hormone; FT3, Free Triiodothyronine; FT4, Free Thyroxine.

Spearman’s correlation analysis (**Table 2**) showed univariate associations between skeletal MM, FT3, FT3 to FT4 ratio, and all investigated variables. MM parameters, including FFM, MM, BCM, and ASMM, resulted directly associated with FT3 levels and the FT3 to FT4 ratio. Moreover, MM was found to be inversely associated with age (P = 0.003, R = −0.196), total (P = 0.035, R = −0.14) and HDL-cholesterol (P < 0.01, R = −0.422) serum levels. By contrast, MM showed a direct relationship with BMI (P = 0.01, R = 0.171), WC (P < 0.01, R = 0.363), insulin-resistance (HOMA-IR) (P = 0.001, R = 0.221), triglycerides (P < 0.01, R = 0.238), uric acid (P < 0.01, R = 0.424), insulin (P = 0.001, R = 0.21), FT3 (P = 0.006, R = 0.18) serum levels, and FT3 to FT4 ratio (P = 0.023, R = 0.151).

**Table 2:** Spearman’s correlation rank matrix between MM, FT3, and FT3 to FT4 ratio and other continuous collected variables.

	MM		FT3		FT3 to FT4 ratio	
	Rho	*P* Value	Rho	*P* Value	Rho	*P* Value
Age (years)	−0.196	**0.003**	−0.049	0.459	−0.011	0.87
BCM (Kg)	0.768	**<0.01**	0.169	**0.011**	0.174	**0.009**
FM (Kg)	0.103	0.12	0.049	0.466	0.019	0.775
FFM (Kg)	0.793	**<0.01**	0.153	**0.022**	0.116	0.081
TBW (L)	0.808	**<0.01**	0.152	**0.022**	0.138	**0.038**
ECW (L)	0.709	**<0.01**	0.106	0.11	0.081	0.226
ICW (L)	0.747	**<0.01**	0.136	**0.041**	0.148	**0.026**
MM (Kg)	1	NA	0.18	**0.006**	0.151	**0.023**
ASMM (Kg)	0.914	**<0.01**	0.181	**0.006**	0.162	**0.015**
BMI (Kg/m^2^)	0.171	**0.01**	0.054	0.42	0.071	0.284
WC (cm)	0.363	**<0.01**	0.077	0.247	0.111	0.095
SBP (mmHg)	0.124	0.063	0.083	0.214	−0.001	0.988
DBP (mmHg)	0.13	0.051	0.051	0.442	−0.006	0.924
Triglycerides (mg/dl)	0.238	**<0.01**	0.13	0.051	0.174	**0.009**
HDL Cholesterol (mg/dl)	−0.422	**<0.01**	−0.077	0.25	−0.104	0.117
LDL Cholesterol (mg/dl)	−0.074	0.266	0.069	0.297	0.126	0.058
FBG (mg/dl)	0.089	0.182	0.006	0.926	0.071	0.286
Insulin (UI/ml)	0.21	**0.001**	0.111	0.095	0.223	**0.001**
HOMA-IR	0.221	**0.001**	0.095	0.152	0.215	**0.001**
Total Cholesterol (mg/dl)	−0.14	**0.035**	0.046	0.494	0.11	0.099
Uric Acid (mg/dl)	0.424	**<0.01**	0.112	0.092	−0.037	0.58
TSH (mU/L)	−0.092	0.166	0.055	0.409	0.171	**0.01**
FT3 (pg/ml)	0.18	**0.006**	1	NA	0.617	**<0.01**
FT4 (pg/ml)	−0.046	0.494	0.165	**0.013**	−0.615	**<0.01**
FT3 to FT4 ratio	0.151	**0.023**	0.617	**<0.01**	1	NA

Statistical significance shown in bold.

PhA, Phase Angle; BCM, Body Cell Mass; FM, Fat Mass; FFM, Free-Fat Mass; TBW, Total Body Water; ECW, Extracellular Water; ICW, Intracellular Water; MM, Skeletal Muscle Mass; ASMM, Appendicular Skeletal Muscle Mass; BMI, Body Mass Index; WC, Waist Circumference; SBP, Systolic Blood Pressure; DBP, Diastolic Blood Pressure; FBG, Fasting Blood Glucose; HOMA-IR, Homeostasis Model Assessment for Insulin Resistance; TSH, Thyroid-Stimulating Hormone; FT3, free triiodothyronine; FT4, Free Thyroxine.

Three sets of linear multiple regression models were built on the FT3 to FT4 ratio in order to investigate the association with MM (**Table 3**). The selection of confounders was made considering both the univariate significance with the FT3 to FT4 ratio and major covariates such as age, gender, and BMI. Raw and semi-adjusted models confirmed the relationship between MM and the FT3 to FT4 ratio independently of age, BMI, TSH, triglycerides, and insulin serum levels (P = 0.040, 95% CI 0.0001 to 0.0024). This association was not maintained when gender was entered into the model, as shown in the fully adjusted model (P = 0.312, 95% CI −0.0008 to 0.0025). However, after dividing the sample by gender, the independent relationship was only maintained in men (P = 0.003, 95% CI 0.0009 to 0.0034) (**Table 4**).

**Table 3:** Linear regression models applying the FT3/FT4 ratio as dependent variable.

	Coefficient	Standard Errorr	CI 95%	*P* Value
MM (Kg)	0.0015	0.0006	0.0004 to 0.0027	**0.006**
				
	**Coefficient**	**Standard Errorr**	**CI 95%**	***P* Value**
MM (Kg)	0.0012	0.0006	0.0001 to 0.0024	**0.040**
TSH (mU/L)	0.0048	0.0031	−0.0012 to 0.0109	0.117
Age (years)	0.0001	0.0003	−0.0004 to 0.0006	0.757
BMI (Kg/m^2^)	−0.0002	0.0007	−0.0015 to 0.0011	0.787
Triglycerides (mg/dl)	0.0001	0.0001	−0.0001 to 0.0002	0.244
Insulin (UI/ml)	0.0006	0.0003	−0.0001 to 0.0012	0.084
				
	**Coefficient**	**Standard Errorr**	**CI 95%**	***P* Value**
MM (Kg)	0.0009	0.0009	−0.0008 to 0.0025	0.312
Gender (Men)	0.0068	0.0106	−0.0139 to 0.0275	0.520
TSH (mU/L)	0.0051	0.0031	−0.0010 to 0.0111	0.104
Age (years)	0.0000	0.0003	−0.0005 to 0.0006	0.854
BMI (Kg/m^2^)	−0.0001	0.0007	−0.0014 to 0.0012	0.836
Triglycerides (mg/dl)	0.0001	0.0001	−0.0001 to 0.0002	0.293
Insulin (UI/ml)	0.0006	0.0003	−0.0001 to 0.0012	0.101
				

Statistical significance shown in bold.

MM, Skeletal Muscle Mass; BMI, Body Mass Index; TSH, Thyroid-Stimulating Hormone.

**Table 4:** Linear regression models applying the FT3/FT4 ratio as dependent variable, subdivided by gender.

Women				
	Coefficient	Standard Error	CI 95%	*P* Value*
MM (Kg)	−0.0009	0.003	−0.0016 to 0.0005	0.261
**Men**				
	**Coefficient**	**Standard Error**	**CI 95%**	*P* Value*
MM (Kg)	0.0031	0.005	0.0009 to 0.0034	**0.003**

Statistical significance shown in bold.

*Corrected by age, BMI, TSH, Insulin, and triglycerides.

## Discussion

This study, carried out in a population of subjects with overweight and obesity, was conceived to investigate the hypothesis that a higher MM is responsible for higher FT3 production and circulating levels in a preexisting condition of obesity which, *per se*, triggers increased FT3 concentrations as a possible compensatory mechanism for a thermogenic defect (10). Our findings show, for the first time, that a preserved skeletal MM within a framework of excess weight has a direct influence on thyroid function, in terms of a higher conversion of FT4 to FT3, independently of age, BMI, TSH, triglycerides, and insulin serum levels, but is affected by gender.

As amply described (23, 24), type II iodothyronine deiodinase (DIO2) activity has been shown to be expressed in skeletal muscle cells, thus opening an interesting research perspective on MM involvement in local and systemic T3 production. As the skeletal muscle is a major site for thermogenesis, glucose utilization and lipid oxidation, our findings reflect the possibility of a biological attempt to counteract overfeeding and weight gain by accelerating oxidative processes through the stimulation of thermogenesis. In agreement with this line of thinking, we found a significant correlation between MM and FT3 levels, as well as the FT3 to FT4 ratio, without any significant relationship between MM and FT4 levels. Only a few studies have previously described a similar but opposite mechanism, suggesting that after a period of calorie restriction, our body tends to adopt a thrifty attitude, altering the local muscle metabolism of thyroid hormones and decreasing the availability of T3, thus basically ensuring a kind of quick restoration of fat reserves (25).

Equally conceivable, though needful of further investigations, is the mechanism involving myokines. From this perspective, irisin is widely known to be increased in subjects with obesity (26), and was recently found to be released also by skeletal muscle. In both cases, its major tasks consist of suppressing appetite while increasing energy expenditure so as to slow down weight gain. Noteworthily, leptin was very recently demonstrated to stimulate FT3 *via* activation of the conversion of FT4 to FT3, and this mechanism supports the hypothesis of a crosstalk between thyroid and skeletal muscle mediated by the muscle-myokine irisin (8, 27). Furthermore, our data suggest that gender has a key role in influencing the association between MM and the conversion of FT4 to FT3, since this relationship appeared to be more pronounced in men, who are well-known to have a higher MM than women. This is a clear effect due to the marked physiological discrepancy in sex hormones secretion and adipose tissue metabolism (28, 29). In other words, while women have higher amounts of body fat storage due to the higher energy demands of their reproductive nature, men exhibit a quantitatively higher amount of fat-free body mass, including bone as well as lean body mass.

In addition, consistently with previous findings (10, 29), this study confirms the FT3 to FT4 ratio as an indicator of an unfavorable metabolic profile, given its association with higher serum triglycerides and insulin levels, as well as insulin resistance (HOMA-IR), all factors with the common effect of strengthening the DIO2 activity. Additional findings of a significantly lower MM in older subjects, as well as a higher BMI and WC in subjects with higher MM, strengthen the internal validity of our results. Equally in line with literature data is the descriptive finding of the prevalence of metabolic syndrome; in fact, this has already been described as predominant in males, in a large sample drawn from the Italian population (30).

Of note, our results are in apparent contrast with those of a previous study (31) showing a negative correlation between the skeletal MM index (SMI) and FT3 levels. However, the authors investigated normal weight Asian subjects, without excluding any use of medications, whereas we examined only Caucasian individuals with a BMI of more than 25 and taking no medications. We also applied a different parameter to quantify MM. Thus, there is insufficient basis for a comparison of these conflicting results.

Some limitations of this study should be considered. Because of the cross-sectional setting, the direction of any causal relationship could not be established, and prospective studies are needed to clarify a causal relationship. Moreover, no evaluation of dietary habits was made and, therefore, we cannot establish any effect of previous diet on anthropometrics and metabolic parameters. This may represent a weak point of our study, since caloric intake may influence directly DIO2 pathway and indirectly thyroid hormones (32). It should also be pointed out that BIA tends to overestimate FFM, however, under stable hydration, as in our case, this method may be useful as an alternative to DXA in a selected clinical setting (33). Furthermore, low correlation coefficients, despite being significant, decreased the biological value of correlations, because they were less than 0.5, a well-recognized cut-off (34) to define a scientifically robust correlation, independently of the p value. Lastly, to complete the picture, further research in this field should take into account pre- and post-menopausal status, since the relative drop in estrogen leads to a decrease in bone mass density and MM/strength (35, 36).

By contrast, a strong point is that we examined only individuals taking no medication, thus avoiding a possible interference with outcomes.

In conclusion, this study shows, for the first time, that a preserved skeletal MM in excess weight conditions helps to stimulate thyroid function, by increasing T4 conversion to T3, and hence T3 availability. The phenomenon is more pronounced in men, due to their greater advantage in terms of MM.

## Data Availability Statement

The raw data supporting the conclusions of this article will be made available by the authors, without undue reservation.

## Ethics Statement

The studies involving human participants were reviewed and approved by IRCCS Istituto Tumori Giovanni Paolo II Bari. The patients/participants provided their written informed consent to participate in this study.

## Author Contributions

Conceptualization: GDP and GG. Methodology: RS. Software: FC and RS. Validation: RS. Formal analysis: FC. Investigation: LLa and SP. Resources: GG. Data curation: FC. Writing—original draft preparation: RZ. Writing—review and editing: GDP, VT, LDL, and VG. Visualization: GDP. Supervision: GDP and RS. Project Administration: GG.

## Conflict of Interest

The authors declare that the research was conducted in the absence of any commercial or financial relationships that could be construed as a potential conflict of interest.
